# Splenic Artery Ligation: An Ontable Bail-Out Strategy for Small-for-Size Remnants after Major Hepatectomy: A Retrospective Study

**DOI:** 10.3390/jpm12101687

**Published:** 2022-10-10

**Authors:** Kassiani Theodoraki, Antonios Vezakis, Dimitrios Massaras, Aspasia Louta, Nikolaos Arkadopoulos, Vassilios Smyrniotis

**Affiliations:** 1 Department of Anaesthesiology, Aretaieion University Hospital, National and Kapodistrian University of Athens, 11528 Athens, Greece; 2 Department of Surgery, Aretaieion University Hospital, National and Kapodistrian University of Athens, 11528 Athens, Greece; 3 Intensive Care Unit Department, Aretaieion University Hospital, National and Kapodistrian University of Athens, 11528 Athens, Greece; 4 Department of Surgery, Attikon University Hospital, National and Kapodistrian University of Athens, 12462 Athens, Greece

**Keywords:** hepatectomy, small-for-size syndrome, liver transplantation, ischemia/reperfusion injury, splenic artery ligation, portocaval shunt

## Abstract

It has been reported that the prevention of acute portal overpressure in small-for-size liver grafts leads to better postoperative outcomes. Accordingly, we aimed to investigate the feasibility of the technique of splenic artery ligation in a case series of thirteen patients subjected to major liver resections with evidence of small-for-size syndrome and whether the maneuver results in the reduction of portal venous pressure and flow. The technique was successful in ten patients, with splenic artery ligation alleviating portal hypertension significantly. Three patients required the performance of a portocaval shunt for the attenuation of portal hypertension. Portal inflow modulation via splenic artery ligation is a technically simple technique that can prove useful in the context of major hepatectomies as well as in liver transplantations and the early evaluation and modification of portal venous pressure post hepatectomy can be used as a practical tool to guide the effect of the intervention.

## 1. Introduction

Major hepatectomies may end up with marginal liver remnants, which may prove inadequate to maintain a favorable microcirculatory hemodynamic environment to ensure liver regeneration and hepatocellular metabolic function [1,2]. The mismatching of the remnant volume to the portal vein hemodynamics may end up with the “Small-for-Size Syndrome” (SFSS), characterized by liver dysfunction and occasionally uncontrollable intraoperative bleeding and liver failure [3,4,5].

Extensive experimental and clinical research, mainly in living-related liver transplantation, has highlighted the beneficial effect of temporary or permanent portosystemic shunts to protect the structure and functional liver integrity from the injurious effect of portal overflow [6,7,8]. However, excessive portosystemic blood diversion may be detrimental to the liver remnant, and a variety of portocaval shunts have been pioneered to optimize the portosystemic diversion of blood volume in relation to liver remnant requirements [9,10,11,12,13].

The regulation of portocaval shunts to maintain intrahepatic microvascular resistance above the threshold of triggering liver regeneration but lower to that of generating injurious shear stress and congestion is very difficult to achieve. Therefore, less aggressive approaches to modulate the portal flow should be explored. Ligation of the splenic artery in order to reduce the splanchnic blood volume presented to the liver remnant has emerged as an alternative approach to ameliorate the mismatching of the portal vein overflow to the sinusoidal section of the liver remnant [14,15,16].

Modulations of Portal Venous Pressure (PVP) and Portal Venous Flow (PVF) are practical and effective measures to readjust the portal flow to the diminished microvascular milieu after extensive liver resections [5,8,11,17,18,19,20].

Our study aimed at assessing the changes of PVP and PVF and Hepatic Arterial Flow (HAF) in liver remnants ≤30% of the standard liver volume by reducing portal vein overflow via ligation of the splenic artery.

## 2. Materials and Methods

### 2.1. Patients

After approval by the hospital’s ethics committee (418/18-04-2022), the authors retrospectively reviewed the files of 141 hepatectomy cases operated in our institution between 2006 and 2014 and identified a case series of 13 patients, 7 males and 6 females of mean age 49 years (range 26–66 years), who underwent liver resections of more than four segments, based on the Couinaud classification [21]. Six patients had hepatocellular carcinoma, two cholangiocarcinoma, one metastatic melanoma, two metastatic colon carcinoma, and two metastatic gastrointestinal stromal tumor. The patients were classified as American Society of Anesthesiologists (ASA I-II) and Child–Pugh class A [22,23].

Nine patients underwent an extended right hepatectomy and two patients underwent extended a left hepatectomy, with liver remnants II-III and VI-VII, respectively. Two patients were subjected to resections of V-VIII and II–III with liver remnants I and IV. Although the preoperative volumetry had not predicted marginal liver remnants, the weight of the remaining liver was ≤30% of the standard liver weight in our series. The liver-remnant weight was calculated by subtracting the estimated resected liver weight from the standard liver weight of the patient. The standard liver weight was computed using a somatometric method available in the literature [24].

### 2.2. Surgical Technique

All patients were subjected to liver resection under inflow and outflow vascular control by clamping the hepatoduodenal ligament and the hepatic veins. Staging of the malignancies and liver mapping had been carried out by computed tomography, magnetic resonance imaging, magnetic resonance cholangio-pancreatography, and magnetic resonance imaging angiography. An intraoperative ultrasound determined the transection line and plane to achieve a free tumor margin ≥0.5 cm. The resection was executed sharply, on a nearly bloodless surgical field. Liver reperfusion was commenced by first releasing the back flow of the hepatic veins and then gradually the inflow to the liver remnant, taking into consideration the readings of the PVP.

### 2.3. Hemodynamic Monitoring

PVP measurements were carried out by inserting a 20G polypropylene catheter into the stump of the ligated branch of the portal vein and were recorded after allowing a five-minute stabilization period: (i) before liver resection (T_0_), (ii) after reperfusion of the liver remnant (T_1_), and (iii) after completion of the hemodynamic maneuvering (T_2_) (Edwards Lifesciences LLC, Irvine, CA, USA). The flow volume of the portal vein and the hepatic artery were measured by Doppler ultrasonography imaging using a 5–0 MHz transducer (Aloka Prosound 5500, Aloka Co., Tokyo). Values were expressed as total flow (mL/min) per 100 g of remnant weight. Calculations were aided by reconstruction via the software of Doppler ultrasonography, which used the mean flow velocity and the diameter of vessels. Specifically, an estimation line was set parallel to the direction of the blood flow. Thereafter, the velocity profile was displayed and the average velocity over the cross-sectional diameter of the vessel as well as the flow volume were calculated.

### 2.4. Hemodynamic Maneuvering

When the PVP rose to ≥ 20 mmHg at reperfusion (timepoint T_1_), partial clamping of the portal vein was applied to maintain the PVP at levels ≤ 15 mmHg. At the same time, ligation of the splenic artery was carried out and the anesthetic team was asked to keep the Central Venous Pressure (CVP) ≤ 5 mmHg. Reperfusion was gradually reestablished by three consecutive sessions of alternating intervals of 5 min of partial portal clamping and 5 min of full reperfusion (timepoint T_2_). PVP readings < 20 mmHg at time point T_2_ were considered less injurious to the liver remnant volume as long as liver swelling ceased and hemorrhage was controlled. In three patients, however, even after splenic artery ligation, the PVP remained > 20 mmHg and was accompanied by congestion and hemorrhage of the liver remnant. Based on these circumstances, we had to proceed to a portocaval shunt with a 5 mm graft of polytetrafluoroethylene in one patient and to a portocaval anastomosis using the stumps of the ligated right and left portal veins branches in the remaining two patients. The abdomen was closed as usual, with a drain system close to the cut surface. In all patients, liver function was regularly monitored for the first three weeks.

### 2.5. Statistics

The primary end point of the study was to outline the hemodynamic profile after extended liver sections with liver remnants ≤ 30% of the standard liver, regarding PVP, PVF, and HAF. The secondary point was to assess the effects of reducing the PVF either via ligation of the splenic artery and concomitant conditioning of the liver remnant to the injurious effect of portal overflow with gradual reperfusion increments or via portocaval shunt techniques, which have been extensively evaluated in living related liver transplantations.

Data are expressed as mean ± SD and were assessed by one-way analysis of variance for repeated measures (ANOVA). A value of *p* < 0.05 was considered as statistically significant. Data were analyzed with the SigmaPlot for Windows v.11.0 statistical software (Systat Software, Inc., San Jose, CA, USA).

### 2.6. Results

Measurements in the ten patients where splenic artery ligation was successfully accomplished were as follows: PVP values before liver resection (T_0_) were 7.7 ± 1.6 mmHg, while, after reperfusion of the liver remnant (T_1_), PVP values increased significantly in comparison to T_0_ (25.0 ± 2.2 vs. 7.7 ± 1.6 mmHg, *p* < 0.001). After splenic artery ligation (T2), the PVP decreased significantly compared with T1 (13.5 ± 1.7 vs. 25.0 ± 2.2 mmHg, *p* < 0.001), (Figure S1).

Initial measurements of the PVF before liver resection (T_0_) were 135.5 ± 18.1 mL/min/100 g, while after reperfusion of the liver remnant (T_1_) increased significantly in comparison to T_0_ (528.1 ± 141.5 vs. 135.5 ± 18.1 mL/min/100 g, *p* < 0.001). After splenic artery ligation (T_2_), PVF values decreased significantly compared with T1 (319.5 ± 54.9 vs. 528.1 ± 141.5 mL/min/100 g, *p* < 0.001), (Figure S1).

Finally, HAF values were 34.8 ± 6.7 mL/min/100 g before liver resection (T_0_). After reperfusion (T_1_), the HAF decreased significantly in comparison to baseline values (21.8 ± 4.1 vs. 34.8 ± 6.7 mL/min/100 g, *p* < 0.001). After splenic artery ligation (T_2_), HAF values increased in comparison to T2, albeit not significantly (23.3 ± 8.0 vs. 21.8 ± 4.1 mL/min/100 g, *p* = 0.594), (Figure S1).

In three patients, however, even after splenic artery ligation, the PVP remained higher than the cut-off value of 20 mmHg. Based on the accompanying worsening of liver swelling and splanchnic congestion, the performance of a portocaval shunt was deemed necessary, as already described in the methods. After the performance of the portocaval shunt, readings of the PVP in the three patients dropped to 6, 8, and 7 mmHg (from 23, 25, and 22 mmHg, respectively), while readings of the PVF dropped to 150, 175, and 250 mL/min/100 g (from 310, 380, and 350 mL/min/100 g, respectively).

All patients were discharged with mild ascites, which gradually subsided. Kinetics of aspartate aminotransferase, bilirubin, and coagulation showed mild liver dysfunction dissolving over the next three weeks. Chest complications (pleural effusion, pneumonia, atelectasis), biliary fistula, and wound infection were the most notable complications. Postoperative complications in our series were graded according to the Clavien–Dindo classification. According to this, two patients were classified as Grade IIIa, two patients as Grade IIIb, and one patient as Grade V. All other patients were classified as Grade I and II. Patients were also classified according to the International Study Group of Liver Surgery (ISGLS) as follows: five as grade A, seven as grade B, and one as grade C. One of the individuals with a portocaval shunt recovered fully from respiratory and kidney failure, but at the third week succumbed to fatal portomesenteric thrombosis. All other patients were finally discharged from hospital with no need for any rehospitalization within one month after surgery.

## 3. Discussion

In our case series, by the early measurement of portal vein pressure, we identified portal hyperperfusion as a cause of potential small-for-size liver remnant dysfunction and we applied splenic artery ligation as a technically simple procedure to manage the situation. The portal pressure and flow were reduced immediately after the maneuver in ten of the patients, therefore we recommend their measurement in cases of marginal liver remnants so that alleviating measures can be undertaken. In three patients, however, the performance of a portocaval shunt was deemed necessary since, despite splenic artery ligation, there was persistent severe congestion and hemorrhage from the cut surface.

Hepatic malignancies often require extensive hepatectomies, and the residual liver remnant may be suboptimal and exposed to the SFSS under the new hemodynamic mismatching between the relative portal overflow and the diminished sinusoidal capillary microcirculatory section [2,3,4,5]. In other words, when a large volume of liver parenchyma is resected, the microcirculatory sector diminishes and portal flow may prove excessive for the residual liver. Portal overperfusion exposes the lining of the multicellular structure of the capillary and sinusoidal network to shear forces, which inflict structural and functional disorder and end up with what is defined as the SFSS, affecting both remnants in donors and grafts in recipients [3,4]. The structural damage is characterized by badly disrupted sinusoidal and endothelial lining with scattered cells into nearby spaces, while the space of Disse becomes deformed and obliterated with cellular debris, adversely affecting the liver remnant function [1].

The syndrome has been extensively evaluated in clinical and experimental studies to elucidate all the implicated causative factors that may be valuable in formulating an algorithmic profile of hemodynamic variables that could be modified accordingly, to ensure safe cut off values of PVP, PVF, and HAF at a given small-for-size liver graft or liver remnant after right liver donation or extended liver resection [5,8,11,17,18,19,20]. Therefore, the importance of the early evaluation of PVP after hepatectomy has emerged as an independent predictor of postoperative outcome and various strategies have been proposed aiming at modulating portal overflow, which appears to be the main contributor to the SFSS.

In this context, portocaval shunts have been recommended by experts working in liver transplant centers and have been associated with satisfactory graft survival [6]. However, these techniques may also have an inhibitory effect on liver regeneration post hepatectomy, which may limit their use. In fact, it is very difficult to accurately regulate the diverted blood volume without depriving the liver remnant from the regenerative role of enhanced intrahepatic resistance, since high portal flow and pressure are considered important triggers of liver regeneration [25,26]. Additionally, the long-term consequences of portocaval shunts, namely the deprivation of the portal route from the graft occasionally leading to the need for early closure, cannot be overlooked [6].

In our series of patients, we attempted splenic artery ligation as a technique to address portal venous overpressure, taking into consideration the contribution of the splenic artery-derived blood perfusion to the total portal inflow pressure. In fact, an average reduction in portal flow of 52% after splenic artery ligation has been reported [27]. We were able to prophylactically modulate portal inflow through splenic artery ligation since we managed to reduce the PVP and the PVF with this technique. The rise in the hepatic arterial flow after splenic artery ligation reflected the arterial buffer response after the decrease in the portal venous blood flow. We were also able to demonstrate that both PVP and PVF are practical tools that can be used in order to perform hemodynamic modulations, thus ensuring an intrahepatic microcirculatory environment less injurious for the cellular structure of the sinusoidal space [18]. This was manifested by the gradual decrease in aspartate aminotransferase levels and the improvement in the coagulation profile over the course of the following three weeks. In the three patients in whom splenic artery ligation did not manage to reduce portal vein pressure and flow, it is possible the situation was aggravated by a concomitant outflow obstruction leading to severe liver and gut congestion and a persistent hemorrhage of the liver gut surface, thus necessitating the performance of a portocaval shunt.

Our findings are in accordance with reports highlighting the favorable impact of a splenectomy or splenic artery ligation on the outcome of recipients of both right and left-lobe grafts in living-donor liver transplantation. A case of primary nonfunction after a small-for size right lobe living-donor liver transplant was salvaged by the reduction of portal pressure and flow after the splenic artery ligation [14]. In another study, the beneficial impact of a splenectomy or splenic artery ligation on the outcome of a living-donor liver transplantation using left lobe grafts was demonstrated [16]. In fact, in the latter study, in a large number of patients subjected to a splenectomy, the portal pressure as well as the portal vein flow after the splenectomy decreased in comparison to that before the splenectomy. In another report, preoperative splenic artery embolization resulted in intraoperative portal decompression, minimized the risk of bleeding during the perioperative period, and, by reducing the graft perfusion pressure to safe levels, ensured satisfactory liver function after liver transplantation [28]. In another study by the same authors, prophylactic splenic artery modulation, either by embolization or ligation in live-donor liver transplantation, relieved excessive portal flow and improved postoperative clinical indices, without untowardly affecting postoperative liver regeneration [29]. Therefore, the attenuation of portal inflow by splenic artery manipulation may prevent portal hyperperfusion injury via attenuation of portal inflow and, in fact, may facilitate liver regeneration.

Additionally, there are a few experimental studies exploring the effect of splenic artery ligation in the context of a major hepatectomy. Splenic artery ligation in combination with an 85% hepatectomy in a murine model induced a relative decrease in oxidation markers during the first 48 h compared with controls subjected to a hepatectomy without splenic artery ligation [15]. Additionally, it promoted an increase in hepatocellular viability and regeneration, without impairing hepatic function. Ito et al., showed that splenic artery ligation improved the remnant liver function in partially hepatectomized rats subjected to ischemia/reperfusion injury [30]. Specifically, splenic artery-ligated animals had lower postoperative aspartate aminotransferase levels and a higher recovery of liver remnant weight, prompting the authors to conclude that these favorable effects may be mediated at least in part via the reduction in portal pressure. In another study, Irie et al., demonstrated that splenic artery ligation ameliorated hepatic ischemia/reperfusion injury and increased the recovery rate of the remnant liver mass in a similar model of partially hepatectomized rats [31]. The authors postulated that the upregulation of the expression of cytoprotective heme oxygenase-1 underlies the favorable effect of splenic artery ligation.

It appears, therefore, that the decompression of portal inflow through splenic artery ligation tends to protect the remaining liver parenchyma from the injury induced by a hepatectomy, thus decreasing morbidity and improving survival. It could allow operations in patients whose lesions or hepatic dysfunction precludes them from major liver resections and could increase the extent of resection in metastatic liver disease, allowing the reduced use of blood products and shorter hospitalizations with obvious advantages in survival rates. In the field of liver transplantation, it could allow the use of smaller liver grafts, increasing the donors’ available pool, without putting the patients at risk for SFSS. It also appears that the technique has distinct advantages compared with the alternative of splenectomy. The reason is that the ligation of the artery close to its origin blocks the arterial blood flow more effectively. In contrast, when a splenectomy is performed, by ligating the branches of the splenic artery too close to the spleen, there may still be intact flow to the splanchnic circulation via the rich collateral system of the peri-celiac trunk and the superior mesenteric artery, therefore portal hyperperfusion may still persist postoperatively. In addition, splenectomy is a seriously invasive surgical procedure associated with complications, such as an increased risk of hemorrhage. Moreover, adverse events such as increased susceptibility to infection caused by compromised immunity as well as portal vein thrombosis due to blood stasis in the stump of the splenic vein that subsequently could extend to the superior mesenteric and portal vein may occur after a splenectomy.

In conclusion, in our series of patients, we were able to perform portal inflow modulation via splenic artery ligation, thus minimizing portal venous overpressure and preventing the SFSS. We also highlighted the importance of early evaluation and modification of PVP post hepatectomy as a practical tool to guide the effect of the intervention. By using a simple, reproducible, and non-invasive technique in combination with maintaining a low CVP (≤5 mmHg), we allowed gradual conditioning of the liver remnant, achieving a favorable hemodynamic modulation and relieving small liver remnants from the deleterious effect of portal overflow, without compromising subsequent hepatocellular function. Therefore, splenic artery ligation, by allowing the moderate decrease of portal pressure for the prevention or treatment of hyperperfusion injury, is a technically simple procedure that could be added in the armamentarium not only of liver transplantation but also of major liver surgery.

This case series has been reported in line with the PROCESS guidelines [32]. The protocol of the retrospective analysis was registered post hoc at the clinicaltrials.gov website under ClinicalTrials.gov Identifier: NCT05459883.

## Data Availability

The datasets generated during and/or analyzed during the current study are available from the corresponding author on reasonable request.

## Supplementary Materials

The following supporting information can be downloaded at: https://www.mdpi.com/article/10.3390/jpm12101687/s1, Figure S1: PVP, PVF and HAF variation over time. Text S1: highlights.
