# Supplementary material for: Splenic Artery Ligation: An Ontable Bail-Out Strategy for Small-for-Size Remnants after Major Hepatectomy: A Retrospective Study
Source: J Pers Med. 2022 Oct 10;12(10):1687. doi: 10.3390/jpm12101687 (PMC9605094; doi:10.3390/jpm12101687)
Supplement: Supplementary file 1 [file jpm-12-01687-s001.zip › jpm-1917460-supplementary.pdf]

**Highlights**

- Major hepatectomies may end up to marginal liver remnants
- The mismatching of the remnant volume to the portal vein hemodynamics may end up to the "Small-for-Size Syndrome"
- Portal inflow modulation via splenic artery ligation is a technically simple technique
- It has emerged as an alternative approach to ameliorate the mismatching of the portal vein overflow to the sinusoidal section of the liver remnant
- It can prove useful in the context of major hepatectomies as well as in liver transplantations
- Early evaluation and modification of portal venous pressure post hepatectomy can be used as a practical tool to guide the effect of the intervention

**Figure S1**. PVP, PVF and HAF variation over time.

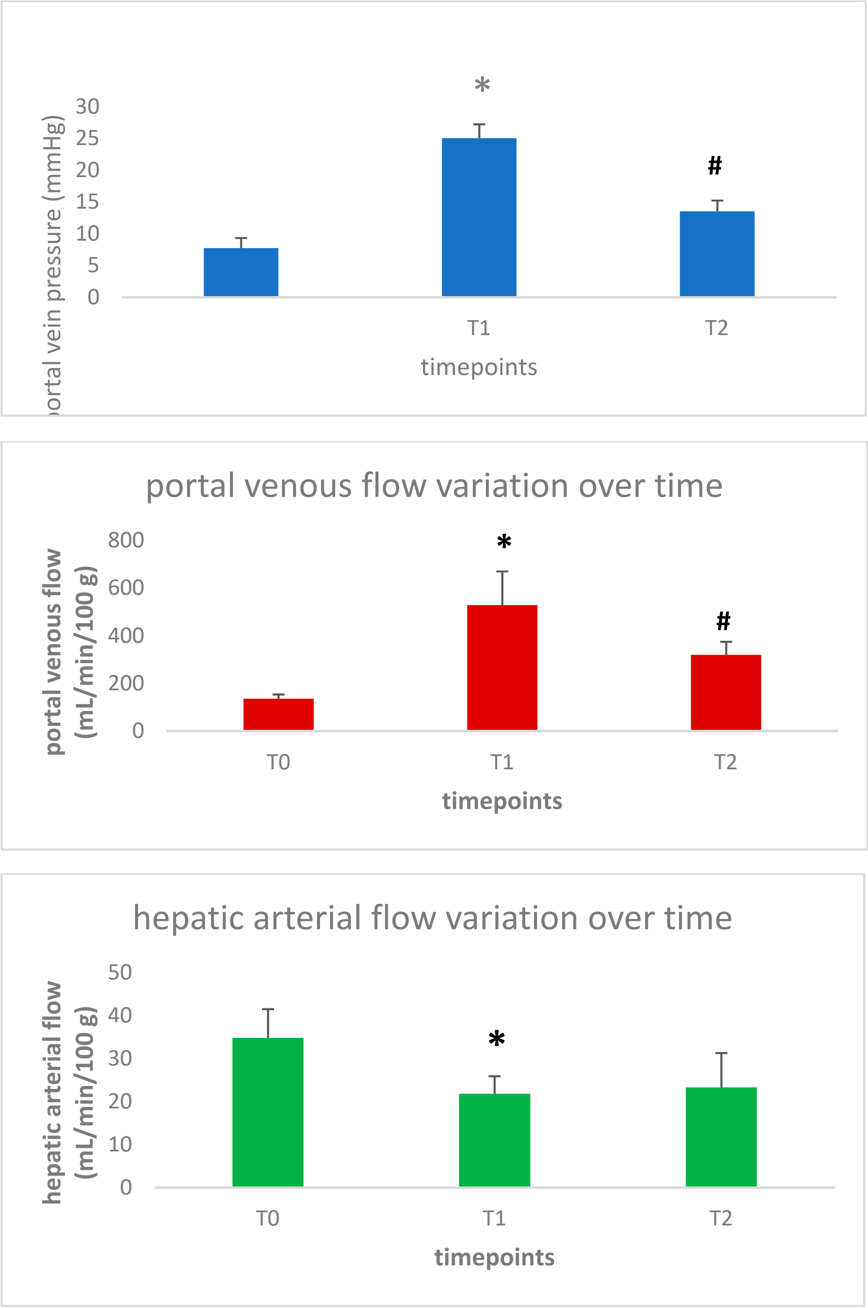


Changes of Portal Vein Pressure (PVP), Portal Vein Flow (PVF) and Hepatic Artery Flow (HAF) before liver resection ($T_0$), after reperfusion ($T_1$) and after splenic artery ligation ($T_2$). Data are depicted as mean (SD). *$p$<0.001 in comparison to T0; #$p$<0.001 in comparison to T1
